# Protective roles of ginseng against bacterial infection

**DOI:** 10.15698/mic2018.11.654

**Published:** 2018-09-19

**Authors:** Ye-Ram Kim, Chul-Su Yang

**Affiliations:** 1 Department of Molecular and Life Science, Hanyang University, Ansan 15588, S. Korea; Department of Bionanotechnology, Hanyang University, Seoul, 04673, S. Korea.

## Abstract

Ginseng (*Panax ginseng* Meyer) is a well-known traditional herbal medicine that plays a protective role against microbial attack. Several studies have revealed its anti-cancer, anti-inflammatory, and immune-modulatory effects. Ginseng contains several components that vary according to the year of cultivation and the processing method used, such as heating, drying, and steaming, which induce different degrees of pharmacological activities. This review discusses the antibacterial effects of ginseng against pathogenic bacterial infections. We describe how ginseng regulates pathogenic factors that are harmful to the host and discuss the therapeutic potential of ginseng as a natural antibacterial drug to combat bacterial infectious disease, which is a global public health challenge. The components of ginseng could be novel alternatives to solve the growing problem of antibiotic resistance and toxicity.

## INTRODUCTION

Because Asia has a specific topography and soil that differs from that of other continents, it provides unique environmental conditions that support the growth of several medicinal plants, which have been used as agricultural products, food, dietary supplements, health supplements, and medicines 1. Botanical medication has been used to treat various diseases in Asia for thousands of years. Among these, ginseng (*Panax ginseng* Meyer, family Araliaceae) is one of the most widely known and used oriental medicinal plants 2. Ginseng is a shade plant that prefers a cool and dry climate, like that of Korea 1. The genus “*Panax*” was named by the Russian botanist Carl Anton Meyer, from the Greek “*pan*” meaning “all” and “*axos*” meaning “cure” 1. The main species of ginseng are *P. ginseng* C.A. Meyer (Korea ginseng), *Panax *quinquefolius** L. (American ginseng), *Panax notoginseng* Burkill (Tienchi ginseng), and *Panax japonicus* C.A. Meyer (Japanese ginseng) 3. By 2009, Korea was the second-highest global producer of ginseng after China 1. Ginseng is globally distributed throughout 35 countries in various forms following processing via drying, steaming, and heating 2.

Several studies have recently reported the beneficial effects of ginseng on diseases such as cancer; immune disorders; diabetes; and liver, neuronal, cardiovascular, and infectious diseases 4,5,6,7,8,9,10,11. Although extracts of ginseng root, leaves, and stems exhibit various pharmacological effects, most pharmacologically active compounds are thought to be present in the root, which has been the focus of previous studies. A significant change in the element accumulation occurs during the life cycle of ginseng. *P*.* ginseng* C.A. Meyer cultivated in Korea is harvested following long cultivation (4-6 years), which allows for the increased composition of secondary metabolites 12. It is consumed after traditional processing methods, including air drying (white ginseng; after 4-6 years' cultivation), steaming and heating (red ginseng; after 6 years' cultivation). Red ginseng is steamed at 98°C-100°C for 2-3 h and then dried at <15% humidity. Because the streaming process enhances its biological activity, red ginseng is more widely used as an herbal medicine than white ginseng 13,14,15. Ginseng contains various bioactive components including tetracyclic triterpenoids (ginsenosides), polyacetylenes, polyphenolic compounds, and acidic polysaccharides of which ginsenoside is highly pharmacologically active.

Although most microorganisms do not induce diseases, some harmful pathogens cause infections in their hosts. When a host is vulnerable to a pathogen, it cannot respond adequately to protect against the infectious disease. Infections are triggered by pathogenic microorganisms, such as bacteria, viruses, parasites, or fungi. The mechanisms of infectious disease development are complex because they depend on interactions between the host, the pathogen, and the environment 16.

Antibiotics are medicines used to prevent and treat bacterial infections. Antibiotic resistance occurs when bacteria change in response to the use of these medicines. Bacteria, not humans or animals, become antibiotic-resistant. These bacteria may infect humans and animals, and the infections they cause are harder to treat than those caused by non-resistant bacteria. Antibiotic resistance leads to higher medical costs, prolonged hospital stays, and increased mortality. Thus, there is an urgent need to re-think the prescription and use of antibiotics. Even if new medicines are developed, without behavior change, antibiotic resistance will remain a major threat. Behavior changes must also include actions to reduce the spread of infections through vaccination, hand washing, practicing safer sex, and good food hygiene 17,18,19. Thus, there is an urgent need to develop novel alternative remedies 20. The pharmacological effects of natural products, especially the antimicrobial activities of plants, are considered to offer attractive novel treatment strategies. Plants interact with various microorganisms and produce small-molecule (<500 Da) antimicrobial compounds that limit the harmful effects of pathogenic microorganisms. Thus, many hundreds of plants have been widely used as traditional medicines 21. Additionally, the combination of natural products and antibiotics exerts a synergistic effect against infectious diseases, resulting in an enhanced antibacterial effect on drug-resistant bacteria and reducing the dosage of existing antibiotics, which alleviates their toxicity 22,23.

Currently, food-related immune system enhancement has attracted attention because of the global emergence of infectious disease epidemics 24. Infections can cause different phenomena depending on the immune system status of the host. Healthy individuals can defend their bodies against a pathogenic invader and remain asymptomatic, but immunocompromized people could acquire a severe infectious disease from the same pathogen. The size of the immunocompromized population is rising because of increasing longevity, changing nutritive conditions of modern people, and the development of long-term cures for various diseases 25. With this increase in infectious disease, ginseng could provide an effective antibacterial treatment. Ginseng has been investigated for its effect on various aspects of disease treatment, especially its role in protection against microbial attack. This review focuses on the effect of ginseng against bacterial infection.

## THE MAJOR COMPONENT OF GINSENG

**Figure 1:**
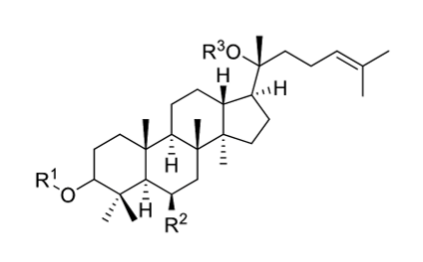
FIGURE 1: General structure of the saponin. Saponins are glucosides with foaming characteristics. Saponins consist of a polycyclic aglycones attached to one or more sugar side chains. The aglycone part, which is also called sapogenin, is either a steroid (C_27_) or a triterpene (C_30_). The foaming ability of saponins is caused by the combination of a hydrophobic (fat-soluble) sapogenin and a hydrophilic (water-soluble) sugar part (R_1_, R_2_, R_3_: -sugar or -H group).

Ginseng comprises saponin and non-saponin constituents. Saponins are glycosides attached to either a saccharide or non-saccharide component (sapogenin and aglycone) (Fig. 1). Ginsenosides refer specifically to ginseng saponins, named to distinguish them from the saponins of other plants. Ginsenosides are specific secondary metabolites of* Panax* sp. and comprise the major pharmacological component of the ginseng plant. Over 30 ginsenosides have been isolated and identified in raw or processed ginseng. Ginsenosides are classified as dammarane or oleanane type, depending on their aglycone skeleton. Dammarane-type ginsenosides, the dominant ginsenoside, are protopanaxadiols (PPDs), protopanaxatriols (PPTs), or ocotillols. PPD-group saponins comprise ginsenosides Ra1, Ra2, Ra3, Rb1, Rb2, Rb3, and Rd; quinquenosides R1 and Rs1-Rs3; and malonyl ginsenosides Rb1, Rb2, Rc, and Rd. PPT-group saponins include ginsenosides Re, Rf, Rg1, Rg2, Rh1, and F1 and notoginsenosides R1 and R2. The ocotillol-group ginsenosides, identified in *Panax* species such as *P*. *quinquefolius*, *P. japonicus*, and *Panax vietnamensis, *comprise majonoside R2 and pseudoginsenoside F11. Ginsenoside Ro has only been identified among the oleanane-group saponins and is a minor component of* P. ginseng *12.

Korean ginseng contains dammarane-type ginsenosides and a unique saponin found in the* Panax *genus that is nontoxic and displays antibacterial activity against non-hemolytic bacteria*. *32 ginsenosides have been isolated from Korea Red Ginseng (KRG), whereas 22, 13, and 14 ginsenoside species have been isolated from white ginseng,* P. quinquefolius, *and *P. notoginseng, *respectively 26. The compound panaxan contains 21 species (R_0_, R_1_, R_2_, R_a_, R_b1_, Rb2, Rc_5,_ Rd_5,_ Re, Rf_5,_ RgI, Rg2, Rg3, RgS_5_ RhI, Rh2, Rh4, RsI, Rs2, Rs3_5_ or R_s4_), and its principal sugar components include glucose, arabinose, galactose, xylose, and rhamnose 27. Several studies have revealed that ginseng components other than ginsenosides also possess pharmaceutical properties. For example, the polysaccharide ginsan, isolated from the ginseng root, can induce physiological effects such as cytokine modulation and lymphoid cell stimulation 28.

## ANTIBACTERIAL ACTIVITY OF GINSENG

Microbial infections cause several distinct diseases, requiring different antibiotic treatments, but inappropriate antibiotic usage triggers antibiotic resistance and leads to various toxic side effects in the host 29. The emergence of multiple-drug-resistant bacteria can render existing antibiotics useless. To address this threat, alternative approaches, such as the use of natural products, have been attempted. This involves targeting bacterial pathways that indirectly kill pathogens and protect the host from bacterial invasions. Such properties have been identified for ginseng 30.

**Table 1:** Summary of the ginseng effects on bacterial infection.

**Microbe**	**Reagent**	**Effect**	**Experimental model**	**Ref**
***H. pylori***	Polysaccharide fractions	Inhibition of hemagglutination	Measurement of hemagglutinating activities, enzyme-linked glacosorbent method *in vitro*	35 36 37
	Fermented ginseng extract containing *L. plantarum* MG 208	Inhibition of cell adhesion, growth and urease activity	Formation of clear zones, measurement of urease activity and cell adhesion activity *in vitro*	38
	RGE	Protective activity against proinflammatory effects in AGS cells	Analysis of cell viability (trypan blue dye exclusion assay, DNA fragmentation assay (comet assay)) Measurement of cytokine level, cell signaling (in vitro)	40
		Polyacetylenes and protopanaxatriol, compounds isolated from RGE inhibit growth in vitro Gineoside Rh1 and protopanaxatriol inhibit H^+^/K^+ ^ATPase	Determination of MICs, Rat gastric H^+^/K^+^ ATPase activity	43
		Suppresses inflammatory mediators	Diet with RGE (200 mg for 6 weeks) in Mongolian gerbils	44
	WGE	anti-*H. pylori* activity *in vitro*	Disc diffusion assay	45
***P. aeruginosa***	Ginseng aqueous extrac	Effect on motility Inhibition of biofilm formation	Motility assays (swimming, twitching motility, swarming), Observation of biofilm formation (confocal laser scanning microscopy)	51
	Dried ginseng	Anti-QS activity	Detection of alginate levels, protease activities, BHL, OdDHL, extracellular proteins	53
		Enhanced Th1 like response, reduced bacterial load in lungs and reduced severity of lung pathology in rats	Effects of ginseng treatment in a rat model Measurement of degree of lung pathology in a mouse model (cytokine level, mortality, CFU, histopathology)	55
		Cytokine modulating effect in a mouse model of *P. aeruginosa* lung infection	CBA/J mice infected *P. aeruginosa* mimics cystic fibrosis patients. Measurement of ginseng effects on degree of severity in a mouse model (cytokine level, mortality, CFU, histopathology)	56
***S. aureus***	KRG	Antibacterial activity *in vitro* (MIC_50_ = 100 µg/mL), Ginsenoside of KRG triggers perturbation of plasma membrane	Antibacterial activity assay, Fluorescent marker calcein leakage measurement from liposomes	60
	Ginsan	Polysaccharide showed anti-septic effects, Ginsan enhanced proinflammatory abilities (NO, proinflammatory cytokine production, phagocytic activity of macrophages). Ginsan modulated TLR pathway	Measurement of survival rate, NO, phagocytic activity, proinflammatory cytokine, CFU in C57/BL6 mice. Analysis of *S. aureus* induced protein-expression, such as TLR, MAPK, NK-κB activation in peritoneal macrophage	61,77
***P. gingivalis***	Polysaccharide	Anti-adhesive activity and anti-hemagglutination	Determination of MIC	67,68
	Heat transformed ginsenoside	Antibacterial activity by damaging bacterial cell membrane integrity	Determination of MIC, cell integrity	69
***L. monocytogenes***	Ginseng extracts from stems and leaves	Antibacterial activity by damaging bacterial cell membrane integrity	Determination of MIC by agar well diffusion assay cell integrity by TEM	72
	KRG	Antimicrobial activity	Paper disc methods Determination of MIC	71
***B. cereus***	KRG	Antimicrobial activity	Paper disc methods Determination of MIC	71
	Fine ginseng root	Antimicrobial activity	Disc diffusion method	75
	Heated ginseng extract	Antimicrobial activity	Disc diffusion method Determination of MIC and MBC	65
***S. pneumoniae***	KRG	Antibacterial activity Protective role against *S. pneumoniae*-induced sepsis *in vivo* Reduced TLR/NK-κB signaling activity *in vitro*	Measurement of survival rate, body weight change, colonizing bacteria	76

Abbreviations: KRG: Korean red ginseng, MBC: minimum bactericidal concentration, MIC: minimum inhibitory concentration, RGE: red ginseng extract, TEM: transmission electron microscopy.

**Figure 2:**
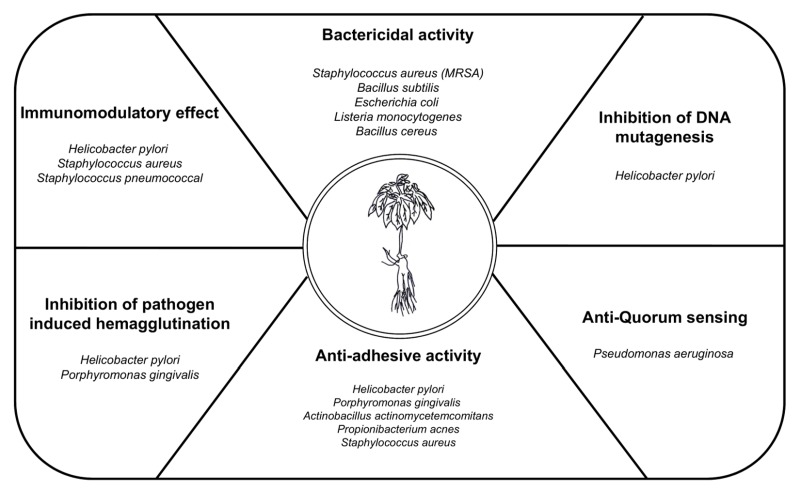
FIGURE 2: The main types of antibacterial activities of *Panax ginseng*. *P. ginseng* shows various antibacterial activities against infectious agents. *P. ginseng* convers antimicrobial therapy via various mechanisms, including bactericidal activity, immunomodulatory effects, inhibition of pathogen-induced hemagglutination, anti-adhesive activity, and anti-quorum sensing activity.

Ginseng indices bactericidal activity, inhibition of DNA mutagenesis, anti-quorum sensing, anti-adhesive activity, inhibition of pathogen-induced hemagglutination, immune-modulatory functions and demonstrates a protective role against pathogen-induced inflammation. The next sections describe the antibacterial effect of ginseng on several representative pathogens (Table 1 and Fig. 2).

### Helicobacter pylori

*Helicobacter pylori (H*.* pylori) *is a highly motile, Gram-negative, microaerophilic bacterium that can infect the stomach, impacting human health 31. *H*.* pylori *infects 50% of the world's population. Most infections are asymptomatic, but some people demonstrate an improper response to the pathogenesis of *H*.* pylori*, developing peptic ulcers, gastric cancer, or malignant lymphoma 32. *H*.* pylori* colonizes the epithelial surfaces of the stomach mucosa in individuals with active chronic gastritis.

The adhesion of pathogenic bacteria to host cells is crucial to initiate host infection, allowing entry into cells beyond the host barrier and subsequent pathogen multiplication 33. Recent research has shown that ginseng extract inhibits cell adhesion, thereby blocking the initiation of pathogenic infections. Pectin-type polysaccharide PG-F2 isolated from *P*.* ginseng* possesses a marked anti-adhesion activity against many microbes 34. Furthermore, ginseng protects against pathogen-induced DNA damage and regulates cell death, as observed in *H*. *pylori* infection.

As *H. pylori* induces gastric inflammation, ulceration, and DNA damage, it has been defined as a class I carcinogen by the WHO. *H*.* pylori *is the most extensively studied and well-known pathogen which is affected by ginseng via various pathways. Acidic polysaccharides from the roots of *P*.* ginseng* C.A. Meyer (Araliaceae) and the leaves of *Artemisia capillaris* (Asteraceae) exert anti-adhesive effects on *H. pylori* infecting human gastric cells and erythrocytes 35,36,37. In recent studies, fermented ginseng extracts, containing the lactic acid bacterium *Lactobacillus plantarum *MG 208, exhibited powerful anti-*H*.* pylori* activity, including anti-bacterial, anti-adhesion, and urease inhibition effects. This extract contained a larger concentration of Rd and R1 ginsenosides compared with other fermented ginseng extracts, which explains its stronger antibacterial activities 38.

Other studies have demonstrated that red ginseng extract (RGE) exerts a protective effect against cytotoxicity and DNA mutagenesis induced by *H*.* pylori* and can reduce proinflammatory activity in gastric mucosal cells. An RGE pretreatment of <100 µg/mL induces protective effects in gastric epithelium cells. RGE also inhibits DNA damage and apoptosis induced by *H*.* pylori* by inhibiting ERK1/2 signaling. This reduces caspase-3 activation and subsequent programmed cell death, consequently diminishing proinflammatory cytokine IL-8 or 5-lipoxygenase mRNA expression 39,40,41,42. A study by Bae *et al*. (2001) revealed the inhibition of *H*.* pylori *growth by polyacetylenes and PPT isolated from RGEs. Moreover, panaxytriol was shown as partially effective in inhibiting *H*.* pylori *growth (MIC_50_ = 50 µg/mL). Ginsenoside Rh1 and PPT can cause minor inhibitory effects on H^+^/K^+^ ATPases, which are involved in the final step of stomach acid secretion 43. In an animal model using Mongolian gerbils, RGE showed inhibitory effects on *H*.* pylori*-induced inflammation. One week after infection with *H*.* pylori* via intra-gastric inoculation, the control diet gerbil group was compared with gerbils given a diet containing 200 mg RGE for 6 weeks. Although the RGE diet supplementation did not affect the viable *H*.* pylori* count in the stomach, RGE inhibited induction of inflammatory mediators (KC, IL-1β, iNOS, MOP activity, and LPO level) in the gastric mucosa of the gerbils 44.

White ginseng extract (WGE) has also demonstrated anti-*H*.* pylori* activity, cytotoxicity, and anti-inflammatory activity *in vitro*. The antibacterial activity of WGE against *H*.* pylori* was measured using a disk diffusion assay, and it was concluded that the growth inhibition was dependent on the WGE dosage. Additionally, WGE exerts a cytotoxic effect on various human cancer cell lines such as A-549 (human lung carcinoma), HEC-1-B (human endometrial adenocarcinoma), and HeLa (human uterine adenocarcinoma), but not normal cells. Also, an analysis of anti-inflammatory activity using RAW 264.7 cells showed a reduction of nitric oxide (NO) production by WGE treatment 45.

### Pseudomonas aeruginosa

*Pseudomonas aeruginosa* (*P*.* aeruginosa*) is a common environmental Gram-negative, rod-shaped bacterium that is an opportunistic bacillus. *P*.* aeruginosa *can colonize under various conditions by utilizing many environmental compounds as energy sources 46. Infections are common in individuals with cystic fibrosis, thermal injury, chronic wounds, chronic obstructive lung diseases, and urinary tract infections, and in immunocompromized patients with acquired immune deficiency syndrome (AIDS) and AIDS-related complex 39,47,48,49. Because *P*.* aeruginosa* can form biofilms, treatment with antibiotics or via the host immune system is challenging. Thus, *P*.* aeruginosa* has acquired resistance to many antibiotics 50, and therapeutics directly targeting biofilms are required to eliminate *P*.* aeruginosa* infection. In a 2011 study, Hong *et al*. showed that although aqueous ginseng extract did not directly affect the growth of *P*.* aeruginosa*, it reduced biofilm formation *in vitro* 51.

Some pathogenic bacteria use quorum sensing (QS), a cell-to-cell communication mechanism, during the infectious process. QS responds to changes in cell-population density and regulates gene expression systems, and is crucial for establishing an infection. Through QS, bacteria produce and release signaling molecules called autoinducers that affect bacterial behavior based on cell density. Bacteria present in biofilms, surface-attached groups of microbial cells enveloped in an extracellular matrix, communicate with others in the biofilm by synthesizing and reacting to these chemical signals 52. Bacterial biofilms can cause chronic infections by limiting the effectiveness of antibiotics. Thus, biofilm reduction is vital in infectious disease treatment. Susceptibility tests with *in vitro* biofilm models have demonstrated that antibiotics are only effective against bacterial biofilms at concentrations hundreds or even a thousand times the minimum inhibitory concentration (MIC) measured in suspension culture 53,54. Ginseng has demonstrated anti-QS activity by suppressing the efficacy of virulence factor production which is related to QS control 53 and inhibition of biofilm formation 39. Recent research has revealed that QS in *P*.* aeruginosa* is required for biofilm formation 52. *P*.* aeruginosa* pathogenesis is related to QS through the formation of various extracellular virulence factors and biofilms. Therefore, QS could be a novel target for the treatment of *P*.* aeruginosa* infections. A 6-year-old dried form of ginseng reduced the levels of QS signaling molecules such as N-butanoyl-L-homoserine lactone and N-3-(oxododecanoyl)-L-homoserine lactone. These signaling molecules are critical components that induce the production of virulence factors 53. A motility test has demonstrated that ginseng activated swimming and twitching motilities but reduced swarming motility, which is essential for biofilm development 51. The effect of ginseng treatment on *P*.* aeruginosa* pneumonia in an animal model promoted a Th1-like response, which might activate the phagocytes and NK (natural killer) cells, leading to improved bacterial clearance in the lungs which results in a reduced severity of lung pathology and an easier control of the bacterial infection 55,56.

### Staphylococcus aureus

*Staphylococcus aureus* (*S*.* aureus*) is a Gram-positive commensal bacterium and major pathogen that can trigger severe clinical infections. It is widely distributed globally and is strongly resistant to the natural environment. *S*.* aureus* colonizes one-third of the human population and commonly exists on the skin and nasal surfaces of healthy people. This bacterium can colonize nares, skin, and the respiratory tract and invade the skin, tissue, and the bloodstream. When *S*.* aureus* infects the skin, it causes abscesses, sinusitis, and food poisoning. Following bloodstream invasion, *S*.* aureus* replicates and disseminates throughout the body, triggering severe infections such as sepsis and endocarditis 57. *S*.* aureus* is able to build biofilms and is a major antibiotic-resistant pathogen. Therefore, the treatment of *S*.* aureus* infections is critical. Although the development of new antibiotics is progressing, *S*.* aureus* acquires effective resistant mechanisms to antibiotics at a rapid rate. Antibiotic-resistant *S*.* aureus *includes two types, namely, methicillin and vancomycin resistant strains. Methicillin-resistant *S*.* aureus* (MRSA) is resistant to methicillin and other beta-lactam antibiotics, including cephalosporin, ampicillin, and nafcillin, and to almost all antibiotics, which makes treatment of *S*.* aureus*-infected patients complicated 58,59. Ginsenoside extracted from KRG, with an MIC_50_ of 100 μg/mL, has shown antibacterial activity against Gram-positive and Gram-negative bacteria including MRSA and exhibits a similar killing effect as propionic acid, which is a well-known bactericidal agent against MRSA. To identify the antibacterial activity of ginsenoside, a bacterial membrane mimic liposome containing fluorescent marker was used. Treatment with ginsenosides induced the acceleration of fluorescent dye leakage, indicating that ginsenoside disturbs bacterial membranes, thereby causing an antibacterial effect. Combination therapies of antibiotics with ginsenoside have been employed to expand the usage of antibiotics and to prevent the development of resistant strains. The combined effect of ginsenosides and the commercial antibiotics kanamycin and cefotaxime against MRSA has been investigated, and it was concluded that these combinations exerted a synergistic effect against MRSA 60.

Ginsan, a polysaccharide isolated from *P*.* ginseng*, has induced increased NO production and potent phagocytic activity by macrophages. Ginsan stimulation of the macrophages has enhanced anti-septicemic activity and increased the production of proinflammatory cytokines. Additionally, ginsan treatment has increased proinflammatory cytokine production in the murine fibroblast cell line L929 61. Furthermore, Ginsan has demonstrated anti-septicemic activity in mouse models. Ginsan has enhanced survival rates and reduced bacterial burden in the blood during *S*.* aureus*-infected sepsis in mice. Moreover, a combination of ginsan and vancomycin induced higher protective effects than the respective single treatments, as measured by mice survival rates 61,62,63. These results suggest that ginsan possesses a potent anti-septicemic activity by stimulating macrophages and acting as an immunomodulator against sepsis caused by *S*.* aureus *infections *in vitro* and* in vivo.*

The underlying mechanisms of ginsan include its anti-septic activity, affecting the toll-like receptor (TLR) pathway. Ginsan treatment has been shown to reduce proinflammatory and anti-inflammatory cytokine production in *S*.* aureus*-infected mice, and ginsan treatment of peritoneal macrophages stimulated by *S*.* aureus *has suppressed the expression of TLR2, TLR4, TLR9, and the adaptor protein myeloid differentiation primary response 88. Ginsan has also inhibited mitogen-activated protein kinase signaling and NF-κB activation induced by *S*.* aureus *62,63,64*.*

The processing of ginseng using heat transforms its components and has been shown to enhance its antibacterial activity against *S*.* aureus*. The potent antimicrobial compound Rg3, an absent ginsenoside in non-heated ginseng, is produced by heating ginseng at 100°C for 16 h and exhibits a higher antimicrobial activity via a reduction in the cell membrane potential 65.

### Porphyromonas gingivalis

*Porphyromonas gingivalis *(*P*.* gingivalis*) is a Gram-negative, rod-shaped, non-motile, anaerobic, and pathogenic bacterium. It causes periodontal diseases and colonizes the periodontal pocket, gastrointestinal tract, respiratory tract, and colon. This pathogen induces aggressive inflammation which destroys the gingiva supporting the teeth and eventually leads to tooth loss. *P*.* gingivalis* rapidly adheres to and enters host cells to induce proinflammatory cytokines such as IL-1β and IL-6 66. PG-HMW and PG-F2, acidic polysaccharides isolated from the roots of *P*.* ginseng*, have been shown to inhibit the attachment of *P*.* gingivalis* to human oral adenocarcinoma cells such as KB cells 34. Furthermore, PG-F2 has been shown to inhibit *P*.* gingivalis*-mediated hemagglutination. These results suggest that PG-F2 could be developed as the base of a dietary component or as a novel anti-adhesive drug for protection against periodontal diseases 67,68. Additionally, steaming of the American ginseng leaf has been shown to induce conversion from polar ginsenosides to less polar ginsenosides. These heat-transformed saponins easily disturbed cell integrity and exhibited higher antibacterial activity than unprocessed saponins against* P*.* gingivalis* 69.

### Listeria monocytogenes

*Listeria monocytogenes* (*L*.* monocytogenes*) is a facultative pathogenic bacterium that induces listeriosis. It is a small rod-shaped, Gram-positive, facultatively anaerobic bacterium and the most recognized globally virulent intracellular food-borne pathogen. Approximately 20% - 30% of food-borne listeriosis is fatal. It is assumed that *Listeria* triggers 1,600 illnesses in the United States annually, of which 400-500 are fatal 70.

Several procedures have been utilized to extract functional components from ginseng, primarily by using different solvents, such as methanol, ethanol and water 71,72. In a study in 2012, Choi *et al*. showed that a water extract of KRG has demonstrated antibacterial activity against *L*.* monocytogenes *(MIC_50_ = 1.0 mg/mL) but not with an ethanol extract 71. Furthermore, Lee *et al*. showed that ginseng extracts produced from ginseng byproducts, such as stems and leaves, using subcritical water extraction (SWE) have exhibited anti *L*. *monocytogenes* activity. SWE at a high temperature enhanced the extraction yield of the phenolic portion in ginseng stems and leaves and also resulted in higher antibacterial activity. Treatment using a 0.2% of SWE ginseng extract has induced morphological cell damage and the loss of structural integrity of bacterial cell walls. From the results obtained by measuring the leakage of cellular materials through the cytoplasmic membrane during treatment with SWE ginseng extract, it is expected that the antibacterial activity demonstrated against *L*.* monocytogenes* is induced by disrupting membrane integrity 72.

### Bacillus cereus

*Bacillus cereus* (*B*.* cereus*) is a Gram-positive, spore-forming, facultative anaerobe bacterium. It is arranged in chain patterns and is motile because of a flagellum. This pathogen forms heat-resistant spores and can exist in and poison food. *B*.* cereus* is environmentally widespread and has been isolated from soil and plants. It also flourishes in insects and the intestinal tracts of mammals. The bacterium produces many virulence factors, including toxins such as emetic toxin and enterotoxins 73. These toxins can cause two types of illness: one type characterized by diarrhea and the other, called emetic toxin, by nausea and vomiting. These bacteria are present in foods and can multiply quickly at room temperature. The pathogenicity of *B*.* cereus*, whether intestinal or non-intestinal, is associated with the production of a tissue-destructive/reactive exoenzyme. Additionally, food poisoning by intestinal infection triggers a systemic and local infection in immunologically compromised and immunocompetent individuals 74. Treatment with ginseng has been shown to have an antibacterial effect against *B*.* cereus* 71. A study has compared the antibacterial activities among extracts of fine ginseng roots with various solvents. The results revealed that the hexane fraction demonstrated the highest antibacterial activity compared with that of the other fractions 75. Furthermore, the dried stems and leaves of ginseng extract produced by SWE at 190°C have demonstrated antibacterial activity against *B*.* cereus* by causing bacterial cell membrane damage and inhibition of cell growth, as observed using transmission electron microscopy 72. A recent study has shown that heating ginseng enhanced its antimicrobial activity against* B*.* cereus*. Ginseng was extracted using methanol and ethanol and processed at various time points. The antimicrobial activity of the heat-treated ginseng extracts was measured using a disk diffusion method. The results indicated that the ginseng extract heated to 100°C demonstrated the highest antimicrobial activity against *B*.* cereus*. Changes to the ginsenoside composition and contents via the heating process enhanced the bacterial growth inhibition effect 65.

### Staphylococcus pneumoniae

*Streptococcus pneumoniae *(*S*.* pneumoniae*) is a Gram-positive bacterium which is the most common cause of pneumonia, and its infection results in 50% of sepsis cases. Ginsan has demonstrated antiseptic effects against sepsis induced by *S*.* pneumoniae*; a KRG water extract was shown to reduce the severity of pneumococcal sepsis. Upon KRG treatment, mice infected with *S*.* pneumoniae* D39 experienced a smaller reduction in body weight and an enhanced survival rate. Additionally, bacterial colonization was reduced, and lung inflammation decreased following treatment with KRG. Moreover, *S*.* pneumonia-*mediated TLR/NF-κB activation was inhibited by KRG treatment *in vitro* in the same manner as for *S*.* aureus*. KRG increased PI3K-AKT signaling, thereby enhancing cell survival in *S*.* pneumonia*-infected RAW 264.7 cells 76,77.

## CONCLUSIONS AND FUTURE PERSPECTIVES

Several studies have suggested that using ginseng to cure infectious diseases could protect the host against pathogen infection. Ginseng has effects that not only directly kill bacteria but also work against the regulation of bacterial adhesion, inflammation, cytotoxicity, and hemagglutination (Table. 1 and Fig. 2). Although the importance of infectious diseases caused by viruses was recently highlighted, bacterial infections remain the most serious problem. Emerging infectious diseases and antibiotic resistance present an immense global predicament, which is limited by the availability of effective antibacterial agents and vaccines. Additionally, an indiscreet use of antibiotics to solve these infections triggers severe side effects in patients. Because of these problems, natural products like ginseng have been highlighted as treatments for bacterial infection with a verified relatively low toxicity. However, because the causal relationship between specific active components and the bioactivities of ginseng is unclear, additional research is required to understand the use of ginseng as an antimicrobial agent. On the other hand, ginseng research could be applied to the food industry to prevent food poisoning because several food pathogens are affected by the antibacterial activity of ginseng. Additionally, the effect of ginseng byproducts outside the root have largely been ignored, but a recent study has revealed that various portions of ginseng demonstrate biologic effects. Years of cultivation of ginseng are critical because of the accumulation of biologically active ginseng components over time. The processing of ginseng, such as heating, drying, and boiling, transforms these components, enhancing the antibacterial effect of ginseng as shown in some studies. As we have explained, the optimal use of ginseng will require the development of additional studies. Using ginseng as a natural antibiotic could be a powerful way to deal with bacterial infections.
